# Recent advances in polyendocrine metabolic ovarian syndrome, formerly polycystic ovary syndrome, with emphasis on endocrine and metabolic dysfunction and cardiovascular risk

**DOI:** 10.1111/joim.70156

**Published:** 2026-08-30

**Authors:** Maria Forslund, Johanna Melin, Anju E. Joham, Terhi T. Piltonen, D. Aled Rees, Helena J. Teede

**Affiliations:** ^1^ Department of Obstetrics and Gynecology Institute of Clinical Sciences Sahlgrenska Academy University of Gothenburg Gothenburg Sweden; ^2^ Department of Gynecology and Obstetrics Sahlgrenska University Hospital, Region Västra Götaland Gothenburg Sweden; ^3^ Department of Obstetrics and Gynecology University of Helsinki, Helsinki University Hospital Helsinki Finland; ^4^ Monash Centre for Health Research and Implementation Faculty of Medicine Nursing and Health Science Monash University Melbourne Australia; ^5^ Endocrine and Diabetes Units, Monash Health Melbourne Australia; ^6^ University of Oulu Oulu Finland; ^7^ Neuroscience and Mental Health Innovation Institute Cardiff University Cardiff UK

**Keywords:** cardiovascular disease, insulin resistance, polycystic ovary syndrome (PCOS), polyendocrine metabolic ovarian syndrome (PMOS), Type 2 diabetes

## Abstract

Polyendocrine metabolic ovarian syndrome (PMOS), previously known as polycystic ovary syndrome, affects 10%–13% of women of reproductive age and remains underdiagnosed despite its substantial health burden. The introduction of the new PMOS nomenclature in 2026 reflects a fundamental shift in understanding the condition as a lifelong endocrine and metabolic disorder with prominent reproductive, psychological, dermatological, and cardiometabolic consequences. Once considered primarily a reproductive disorder, PMOS is now recognized as a multisystem condition. Insulin resistance and compensatory hyperinsulinemia are central pathophysiological mechanisms linking endocrine and metabolic features to reproductive dysfunction. In PMOS, insulin resistance affects 75% of lean and 95% of overweight women. Here we focus on the metabolic features. Recent advances in epidemiology include the first systematic review and meta‐analysis examining regional variations in PMOS prevalence. In parallel, contemporary population‐based studies of unselected women have confirmed that increased risks of metabolic complications observed in selected clinical cohorts also apply at the population level and have demonstrated increased risk of major adverse cardiovascular events (MACE). These studies report an almost 3‐fold increased risk of Type 2 diabetes (OR 2.9) and MACE (HR 1.2–2.5), with cardiovascular risk diverging from approximately 35 years of age. In this review, we summarize recent advances in epidemiology, pathophysiology, metabolic dysfunction, and cardiovascular risk of PMOS, together with their implications for diagnosis and clinical management. Recognizing PMOS as an early‐life marker of cardiometabolic disease highlights opportunities for earlier diagnosis, prevention, and individualized risk stratification, supporting integrated, patient‐centered care across the life course.

## Graphical abstract

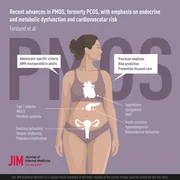

**Abbreviations**
AE‐PCOS: androgen excess polycystic ovary syndrome
AKR1C3: aldo‐keto reductase family 1 member C3
AMH: anti‐Müllerian hormone
CAC: coronary artery calcium
CIMT: carotid intima‐media thickness
COCP: combined oral contraceptive pill
CVD: cardiovascular disease
DHEAS: dehydroepiandrosterone sulfate
DVT: deep venous thrombosis
FMD: flow‐mediated dilation
FSH: follicle‐stimulating hormone
GDM: gestational diabetes mellitus
GLP‐1 RA: glucagon‐like peptide‐1 receptor agonist
GnRH: gonadotropin‐releasing hormone
HA: hyperandrogenism
HDL: high‐density lipoprotein
IFG: impaired fasting glucose
IGT: impaired glucose tolerance
LH: luteinizing hormone
MACE: major adverse cardiovascular events
MASLD: metabolic dysfunction‐associated steatotic liver disease
NIH: National Institutes of Health
NK3R: Neurokinin 3 receptor
OD: ovulatory dysfunction
OGTT: oral glucose tolerance test
PCOM: polycystic ovarian morphology
PCOS: polycystic ovary syndrome
PMOS: polyendocrine metabolic ovarian syndrome
SHBG: sex hormone‐binding globulin
T2DM: Type 2 diabetes mellitus
VLDL: very‐low‐density lipoprotein
WHO: World Health Organization

## Introduction: evolving perspectives on PMOS, including a new name

Polyendocrine metabolic ovarian syndrome (PMOS), formerly called polycystic ovary syndrome, relies on diagnostic criteria in adults, including hyperandrogenism, ovulatory dysfunction (OD), and polycystic ovarian morphology or increased anti‐Müllerian hormone (AMH) levels [1, 2]. The diagnostic criteria capture the downstream most common impacts of hormonal change aligned to contemporary research and guidelines that recognize PMOS as a complex multisystem condition with important metabolic and cardiometabolic implications across the life course [3]. Recent epidemiological, mechanistic, and longitudinal studies have substantially advanced our understanding of PMOS, identifying insulin resistance and hyperinsulinemia as central drivers linking reproductive dysfunction with long‐term metabolic and cardiovascular disease (CVD). Women with PMOS have an approximately 3‐fold increased risk of Type 2 diabetes and a 20%–70% increased risk of CVD, depending on the population studied, with elevated risks for major adverse cardiovascular events (MACE) emerging from early adulthood. Insulin resistance and hyperinsulinemia are considered central components of the underlying pathophysiology, contributing not only to reproductive features but also to adverse metabolic profiles that may emerge early, including in individuals without obesity.

The evolving understanding of this condition has also transformed how PMOS is conceptualized and communicated and intensified the long‐standing debate regarding whether its name accurately reflected contemporary science, patient experience, and clinical practice. International evidence‐based guidelines highlight PMOS as a multisystem condition with implications far beyond the ovary [2], contributing to diagnostic confusion, delayed recognition, and stigma [4]. In fact, there is no increase in pathological ovarian cysts, only arrested follicles [5]. The 2012 NIH Evidence‐based Workshop formally recommended renaming the condition to better align with its pathophysiology and improve education, research, and clinical communication [6], affirmed in the 2023 International Guidelines [2].

Recent global efforts delivered a mandate for change supported by 81% of clinicians and those affected by the condition [4]. The multistage process of change engaged patients, multidisciplinary clinicians, 56 professional societies, and advocacy groups across six world regions, with 22,000 survey responses and multiple international workshops. This large, methodologically rigorous, international process [1, 4] established principles: scientific accuracy, communication clarity, cultural and linguistic appropriateness, and avoidance of stigma [1]. Stakeholders endorsed moving away from the inaccurate “cystic” descriptor and narrow gynecological framing and include the endocrine and metabolic disturbances that characterize the condition [1].

The new name PMOS reflects genetic origins, pathophysiology, and clinical features and represents a significant move toward an updated disease framework. Importantly, this change is evolutionary rather than revolutionary and is supported by a comprehensive 3‐year transition period [1]. Consistent with this shift in terminology, PMOS is expressed as a heterogeneous condition with multisystem features, including core metabolic features. Here we aim to review these in the context of the new name.

### Clinical expression of PMOS

PMOS is associated with endocrine, metabolic, reproductive, dermatological, and psychological features. Contemporary evidence demonstrates a marked cardiometabolic burden, including dyslipidemia affecting 60%–70% of women with PMOS, hypertension occurring 2‐ to 3‐fold more frequently than in controls, and an almost 3‐fold increased risk of Type 2 diabetes (Table 1) [7, 8]. Emerging evidence suggests that these abnormalities may be present from childhood and adolescence and persist across adulthood, supporting the concept of accelerated cardiometabolic aging in this population [9, 10, 11, 12]. Importantly, PMOS is a heterogeneous condition, and metabolic risk varies substantially across ethnicities, populations, body mass index (BMI) categories, and life stages, indicating that a one‐size‐fits‐all model is insufficient to capture its heterogeneity and complexity [3]. Women with PMOS experience substantial psychological morbidity, including 3.8‐fold higher odds of depressive symptoms, 5.6‐fold higher odds of anxiety symptoms, and reduced health‐related quality of life, further emphasizing its multisystem nature [13]. Collectively, these findings demonstrate that the burden of PMOS extends far beyond reproductive dysfunction, encompassing substantial metabolic, cardiovascular, and psychological morbidity across the life course.

**Table 1:** Clinical manifestations and long‐term health consequences of polyendocrine metabolic ovarian syndrome (PMOS).

Domain	Manifestation	Approximate frequency/risk	Key message
Reproductive	Ovulatory dysfunction	90%–95%	Most common cause of anovulatory infertility
Reproductive	Hyperandrogenism	60%–80%	Key pathophysiological feature. Core diagnostic feature
Reproductive	Delayed childbearing	Increased	Despite similar parity in Nordic cohorts
Pregnancy	Gestational diabetes	OR 2.4	Major contributor to pregnancy risk
Pregnancy	Preeclampsia	OR 2.3	Part of the increased risk of hypertensive disorders of pregnancy
Metabolic	Insulin resistance	∼75% (lean); ∼95% (overweight)	Central pathophysiological feature
Metabolic	Type 2 diabetes	OR 2.9	Earlier onset than in controls
Metabolic	Metabolic syndrome	OR 3.4	Particularly associated with obesity
Metabolic	MASLD	Increased	Pooled prevalence 43% in a meta‐analysis, although estimates varied substantially between studies
Cardiovascular	Dyslipidemia	60%–70%	Atherogenic lipid profile
Cardiovascular	Hypertension	2–3‐fold increased risk	Early onset
Cardiovascular	Major adverse cardiovascular events	HR 1.2–2.5	Risk diverges from approximately age 35
Psychological	Depressive symptoms	OR 3.78 (95% CI 3.03–4.72)	Increased independently of BMI
Psychological	Anxiety symptoms	OR 5.62 (95% CI 3.22–9.80)	Increased independently of BMI

Abbreviation: BMI, body mass index; MASLD, metabolic dysfunction‐associated steatotic liver disease.

As illustrated in Fig. 1, these evolving insights have contributed to a shift in conceptual frameworks, from viewing PMOS as an isolated condition affecting women during their reproductive years toward understanding it as a life course disorder. Increasing emphasis is placed on early identification of metabolic dysfunction and on preventive strategies aimed at reducing long‐term risk of Type 2 diabetes mellitus (T2DM) and CVD [2, 14]. A deeper understanding of the metabolic dimension of PMOS is therefore essential to inform risk stratification, guide clinical management, and improve long‐term health outcomes.

**Fig. 1:**
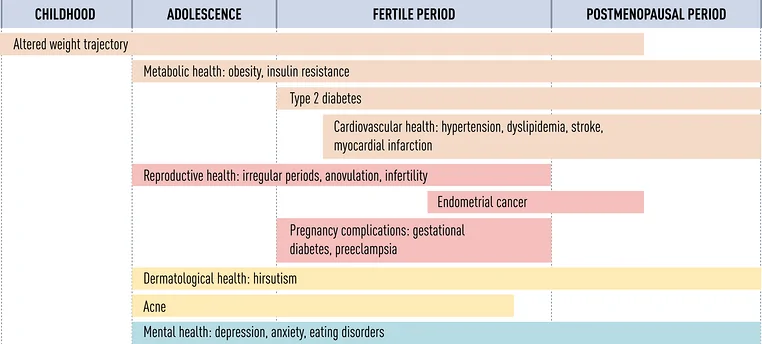
PMOS as a life course disorder: expanding beyond reproductive features. Schematic illustration of the evolving understanding of polyendocrine metabolic syndrome (PMOS) across the life course. Although reproductive manifestations often prompt clinical presentation during adolescence and early adulthood, endocrine, metabolic, cardiovascular, psychological, and reproductive consequences may emerge at different stages of life. The figure highlights the transition from a traditional reproductive‐centered view of the condition toward a life‐course model emphasizing early metabolic dysfunction, cardiometabolic risk, and long‐term health outcomes.

### Diagnosis and prevalence: current perspectives and challenges

PMOS is diagnosed on International Guideline criteria [2, 15], which has built on Rotterdam criteria. In adults, this requires that at least two of the following three criteria are present: (i) clinical and/or biochemical hyperandrogenism; (ii) OD; and (iii) multifollicular ovarian morphology, preferably assessed by transvaginal ultrasound or elevated AMH levels, after exclusion of relevant differential diagnoses [2]. Key differential diagnoses include non‐classic congenital adrenal hyperplasia, hyperprolactinemia, thyroid dysfunction, and ovarian failure, requiring biochemical testing. Other differentials should be excluded clinically and biochemically if indicated, including Cushing syndrome and androgen‐secreting tumors. The current diagnostic criteria [2], key clinical considerations, and recommended exclusion of alternative diagnoses are summarized in Table 2.

**Table 2:** Diagnostic criteria for polyendocrine metabolic ovarian syndrome (PMOS), key clinical considerations, and exclusion of alternative diagnoses.

Diagnostic feature	Current definition	Practical considerations
**Hyperandrogenism**	**Clinical**: Hirsutism assessed using the modified Ferriman–Gallwey (mFG) score, with population‐specific cutoffs (≥4–6; ≥4 in women of Northern European and Asian ancestry). Acne and androgenic alopecia may support the diagnosis but are less specific **Biochemical**: Elevated total testosterone or free androgen index (FAI), preferably measured using high‐quality assays	Clinical and biochemical hyperandrogenism are considered equivalent diagnostic features. Biochemical testing is recommended when clinical findings are equivocal
**Ovulatory dysfunction**	Persistent menstrual irregularity, including menstrual cycles <21 or >35 days, fewer than eight cycles per year, any cycle >90 days, or primary amenorrhea by age 15 years (or >3 years after thelarche)	Menstrual irregularity is physiological during the first year after menarche and should not be used for diagnosis during this period
**Multifollicular ovarian morphology**	**Ultrasound**: Follicle number per ovary ≥20 in at least one ovary is the preferred criterion in adults. When image quality is insufficient or older ultrasound technology is used, ovarian volume ≥10 mL or follicle number per section (FNPS) ≥10 may be used **Alternative biomarker**: Elevated anti‐Müllerian hormone (AMH) may be used instead of ultrasound in adults	Not recommended for diagnosis in adolescents. Transvaginal ultrasound is preferred when imaging is indicated. If transabdominal ultrasound is performed, ovarian volume or FNPS should be used. In women with both hyperandrogenism and ovulatory dysfunction, ultrasound is not required for diagnosis
**Exclusion of alternative diagnoses**	Pregnancy (when appropriate), thyroid dysfunction, hyperprolactinemia, non‐classic congenital adrenal hyperplasia, hypothalamic amenorrhea, and primary ovarian insufficiency should be excluded. Additional investigations for Cushing syndrome, androgen‐secreting tumors, or medication‐induced hyperandrogenism should be guided by the clinical presentation	Initial laboratory evaluation generally includes TSH, prolactin, FSH, and 17‐hydroxyprogesterone. Further investigations should be individualized according to the history and clinical examination

*Note*: In adults, PMOS is diagnosed when at least two of the three diagnostic features are present, following exclusion of alternative endocrine disorders that may mimic the condition. In adolescents, both hyperandrogenism and ovulatory dysfunction are required for diagnosis, whereas multifollicular ovarian morphology and anti‐Müllerian hormone (AMH) are not recommended because of physiological pubertal maturation.

Abbreviation: FSH, follicle‐stimulating hormone.

Diagnosis during adolescence recognized that physiological features of normal pubertal maturation—including irregular menstrual cycles and multifollicular ovarian morphology and elevated AMH levels—overlap with diagnostic features of PMOS, increasing the risk of both over‐ and underdiagnosis [16]. Current International Guidelines therefore recommend that both hyperandrogenism and OD must be present for diagnosis in adolescents, whereas multiple follicles and elevated AMH levels are not considered sufficiently specific in this age group due to the high prevalence of multifollicular ovarian morphology and physiologically elevated AMH levels during normal puberty and early reproductive maturation [2, 16]. Consistent with this, recent meta‐analytic evidence demonstrates that prevalence estimates varied substantially over time as diagnostic criteria evolved, illustrating the dual challenge of potential overdiagnosis when normal pubertal variation is misclassified as PMOS and underdiagnosis when strict criteria fail to capture individuals with clinically relevant symptoms [16]. The International Guideline does not distinguish between possible, probable, or definite PMOS. Rather, diagnosis is either confirmed or not confirmed according to the diagnostic criteria. Adolescents with suggestive features who do not meet full diagnostic criteria are therefore recommended to be considered “at risk” of PMOS and followed longitudinally [2].

Clinical manifestations of PMOS usually emerge during puberty, when menstrual irregularity and hyperandrogenism first become apparent. However, because these features overlap with normal pubertal development, diagnosis is often delayed until late adolescence or early adulthood. Several studies indicate that women frequently experience symptoms for years before receiving a formal diagnosis, representing missed opportunities for early identification of metabolic risk and timely preventive interventions. An Australian study reported that approximately one third of women waited more than 2 years after first seeking medical care before receiving a diagnosis, and nearly half consulted three or more physicians prior to diagnosis [17]. Similar diagnostic delays have been reported in other healthcare systems, reflecting limited awareness of PMOS outside specialist care, variation in clinical practice, and the absence of a single definitive diagnostic test [18, 19, 20]. Importantly, international priority‐setting initiatives emphasize that individuals with PMOS consider timely and accurate diagnosis a major unmet need, highlighting inconsistent application of diagnostic criteria, insufficient information at the time of diagnosis, and the importance of early recognition to support understanding of symptoms and enable preventive management of long‐term metabolic and reproductive health risks [21, 22].

These diagnostic challenges have important clinical implications. Delayed diagnosis may postpone initiation of interventions targeting weight management, lifestyle modification, and metabolic risk reduction, potentially contributing to the early development of obesity, insulin resistance, and T2DM. Conversely, overdiagnosis may expose individuals to unnecessary medicalization and anxiety, particularly during adolescence when physiological variation is common. Improving diagnostic accuracy and timing may therefore represent an important opportunity to optimize both clinical management and long‐term health outcomes.

PMOS is one of the most common endocrine disorders in women of reproductive age, affecting approximately 12% of adult women depending on diagnostic criteria and population characteristics [15]. In adolescents, the prevalence is approximately 6% when current guideline‐recommended criteria are applied, reflecting the challenges of distinguishing PMOS from normal pubertal physiology and reducing the risk of overdiagnosis associated with ultrasound‐based criteria [16]. A substantial proportion of affected individuals remain undiagnosed, as prevalence estimates based on ICD‐coded diagnoses are markedly lower than those observed in population‐based studies, indicating that the true population burden is likely underestimated [15].

Global prevalence varies across geographical regions and ethnic groups. A 2026 global systematic review and meta‐analysis, the first to quantify PMOS prevalence by World Health Organization (WHO) region, demonstrated significant regional variation, with prevalence based on Rotterdam criteria being highest in the Eastern Mediterranean (15.1%) and Southeast Asian regions (14.3%) and lowest in the Western Pacific region (9.1%) [15]. These findings suggest that the burden of PMOS differs across populations and is likely influenced by interactions between genetic background, environmental exposures, and diagnostic practices, highlighting that a uniform global approach may not fully capture disease heterogeneity. Importantly, data remain sparse from several world regions, particularly Africa, highlighting the need for further population‐based studies to improve global estimates and inform future diagnostic and prevention strategies [15].

Although the International Guideline criteria are widely used and are underpinned by endocrine‐driven impacts, metabolic features are not reflected in current diagnostic classification, underscoring the need for improved tools enabling earlier identification of individuals at increased metabolic risk.

### Pathophysiology of PMOS: hormonal and genetic insights

PMOS pathophysiology reflects an intricate interplay among metabolic disturbance, reproductive dysfunction, and endocrine and neuroendocrine dysregulation, superimposed upon polygenic susceptibility and epigenetic factors. There is no single unifying pathogenic mechanism for PMOS; rather, the syndrome arises from the convergence of multiple factors, including perturbations across neuroendocrine, steroidogenic, and metabolic axes resulting in altered function of the hypothalamus–pituitary–ovarian axis. Recent advances increasingly position insulin resistance as the central integrative mechanism linking neuroendocrine dysfunction, androgen excess, and cardiometabolic disease. An overview of the pathophysiology is shown in Fig. 2.

**Fig. 2:**
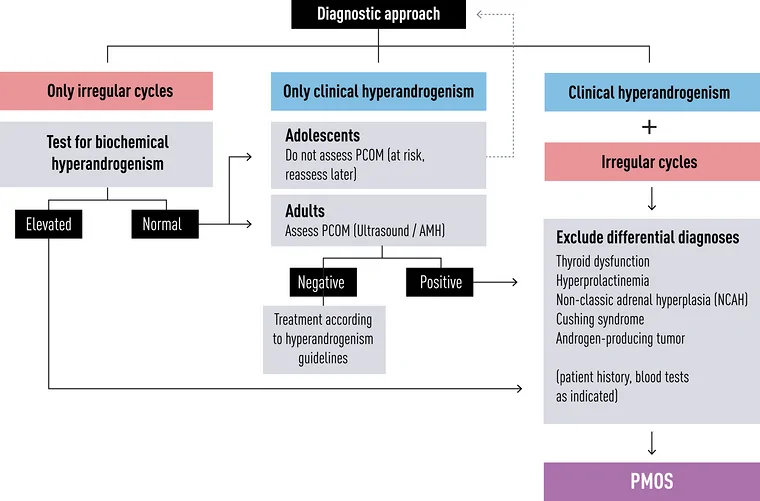
Diagnostic pathway for polyendocrine metabolic ovarian syndrome (PMOS). The diagnostic pathway is guided by the presence of menstrual irregularity and clinical or biochemical hyperandrogenism. In adults, multifollicular ovarian morphology may be assessed by ultrasound or anti‐Müllerian hormone (AMH) when required to establish the diagnosis. In adolescents, ovarian morphology and AMH are not recommended for diagnosis; those with suggestive features who do not fulfill the diagnostic criteria should be considered at risk and reassessed over time. Relevant differential diagnoses should be excluded before PMOS is confirmed. PCOM, polycystic ovarian morphology.

**Fig. 3:**
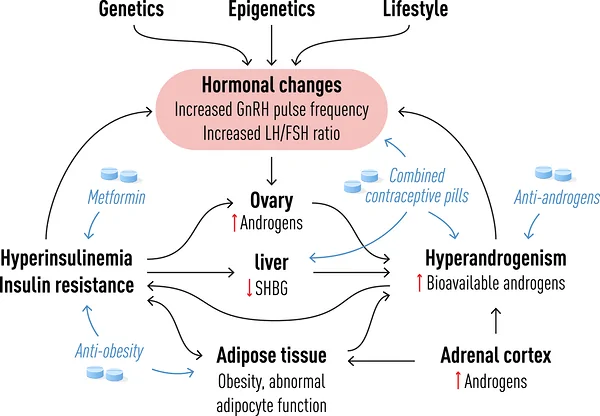
Pathophysiology of polyendocrine metabolic ovarian syndrome (PMOS) and major therapeutic targets. PMOS arises from complex interactions between genetic, epigenetic, neuroendocrine, and lifestyle factors. Insulin resistance and compensatory hyperinsulinemia interact with altered gonadotropin secretion, ovarian, and adrenal androgen excess, reduced hepatic sex hormone‐binding globulin (SHBG) production, and adipose tissue dysfunction, creating self‐reinforcing pathways that promote hyperandrogenism and metabolic dysfunction. Blue arrows indicate the principal targets of commonly used pharmacological treatments. FSH, follicle‐stimulating hormone; GnRH, gonadotropin‐releasing hormone; LH, luteinizing hormone; SHBG, sex hormone‐binding globulin.

#### Insulin resistance and hyperinsulinemia

Insulin resistance constitutes a fundamental pathophysiological feature of PMOS and has been demonstrated to affect 75% of lean and 95% of overweight women using euglycemic–hyperinsulinemic clamps, the gold standard measure of insulin resistance, driven by an intrinsic, obesity‐independent defect that is further compounded by excess adiposity [23]. In PMOS, insulin resistance arises from a distinctive, tissue‑specific molecular defect. Unlike the mechanisms seen in obesity or T2DM, women with PMOS exhibit increased insulin‑stimulated serine phosphorylation of the insulin receptor in skeletal muscle [24, 25]. This abnormal modification disrupts downstream insulin signaling and represents an intrinsic defect that cannot be fully attributed to excess adiposity [24, 25]. The insulin resistance in PMOS leads to compensatory hyperinsulinemia, which drives multiple pathological consequences. It augments hyperandrogenism by acting as a co‐gonadotropin in the ovaries [26], diminishing progesterone‐mediated negative feedback on gonadotropin‐releasing hormone (GnRH) secretion [27], enhancing ACTH‐stimulated adrenal steroidogenesis [28], activating adipocyte aldo‐keto reductase 1C3 (AKR1C3) [28], and suppressing sex hormone binding globulin (SHBG) production [29], collectively increasing circulating bioactive androgens and anovulation [30].

#### Neuroendocrine dysregulation

A central upstream driver of PMOS is increased pulse frequency of GnRH from the hypothalamus, accompanied by diminished sex‑steroid negative feedback at the level of the hypothalamus, resulting in preferential secretion of luteinizing hormone (LH) over follicle‐stimulating hormone (FSH) from the pituitary gland [31, 32, 33]. Downstream, LH stimulates ovarian theca cells to increase androgen production, whereas relative FSH deficiency and increased testosterone inhibit ovarian follicular maturation and selection, leading to follicular arrest and accumulation of many small antral follicles leading to multiple follicles on ovarian morphology [31, 32]. Granulosa cells in the small antral follicles produce excess AMH, further exerting an inhibitory effect on follicle recruitment and maturation [24]. AMH also inhibits the conversion of testosterone to estradiol. In addition, hyperinsulinemia stimulates ovarian theca cells and suppresses SHBG production, further contributing to hyperandrogenemia [3].

The KNDy neuron system—comprising kisspeptin, neurokinin B, and dynorphin A neurons in the hypothalamic infundibular nucleus—is central to regulating this GnRH pulse generator [34]. Neurokinin B acts via Neurokinin 3 receptors to stimulate kisspeptin release, which further stimulates GnRH release, whereas dynorphin exerts inhibitory modulation. In PMOS, circulating kisspeptin levels are elevated, and the normal negative feedback from estradiol and progesterone on GnRH pulsatility is impaired [24, 35]. Because GnRH neurons lack steroid receptors, this feedback operates indirectly through kisspeptin neurons, which express progesterone and estrogen receptors. Elevated androgens in PMOS appear to disrupt this progesterone‐mediated negative feedback, perpetuating abnormal GnRH drive. AMH further amplifies GnRH pulsatility by acting on AMH receptors expressed on hypothalamic neurons, compounding the neuroendocrine dysregulation [36].

#### Hyperandrogenism

Hyperandrogenism is a key feature of PMOS and is detected in 60%–80% of women with PMOS [37]. There is increased production of ovarian androgens from ovarian theca cells, driven by increased secretion of LH, hyperinsulinism, and increased theca cell responsiveness to LH [26, 38]. Beyond the ovaries, the adrenal glands contribute to androgen excess. Elevated dehydroepiandrosterone sulfate is detectable in 20%–30% of women with PMOS [39], and more recently, peripherally generated 11‐oxygenated androgens—particularly 11β‐ketotestosterone and 11β‐ketodihydrotestosterone [40]—have been identified as potent circulating androgens that bind the androgen receptor with potency comparable to testosterone [41]. These are generated in subcutaneous adipose tissue via the enzyme AKR1C3, whose expression is upregulated by insulin [41, 42]. Hyperinsulinemia also suppresses hepatic SHBG production, increasing the fraction of biologically active free androgens [29], which in turn promotes visceral adiposity and adipocyte dysfunction [43], contributing to insulin resistance. These mechanisms perpetuate a cycle that links metabolic dysfunction with androgen excess in women with PMOS.

#### Adipose tissue and metabolic dysfunction

Adipose tissue in PMOS exhibits distinctive morphological and functional abnormalities. Women with PMOS show adipose‑tissue‐specific defects, including reduced insulin‑stimulated glucose uptake due to decreased GLUT4 expression and impaired lipolysis due to fewer β_2_‑adrenergic receptors. Their adipocytes also exhibit abnormal lipid storage and increased intra‐adipose androgen generation via elevated AKR1C3 activity, promoting ectopic lipid deposition in skeletal muscle and liver, as well as systemic lipotoxicity, thereby further contributing to insulin resistance. Inflammation, increased macrophage infiltration, fibrosis, and expansion of adipose tissue worsen insulin resistance in PMOS.

#### Genetic and epigenetic inheritance

PMOS is highly heritable, accounting for ∼70% of the development of the disorder [44], yet known GWAS loci account for only around 10% of this heritability [31]. Of the genes that have been identified, susceptibility variants cluster around neuroendocrine genes (*FSHR*, *LHCGR*, and *FSHB*) and metabolic genes (*INSR*, *THADA*, and *DENND1A*) [24]. Prenatal exposure to excess androgens, to which daughters of women with PMOS are subjected due to elevated maternal androgens and impaired placental aromatase [45], may induce epigenetic modifications in somatic and germ cells [24], a mechanism demonstrated in animal models [46] and supported by emerging human data. In addition, differential DNA methylation patterns in umbilical cord blood of neonates born to mothers with PMOS indicate a distinct epigenetic signature [24, 47], supporting the presence of an adverse maternofetal environment that alters fetal epigenetic programming.

Recent advances suggest that PMOS develops through complex interactions between genetic susceptibility and environmental exposures across the life course rather than a single initiating event [3]. Prenatal androgen excess and an adverse intrauterine environment may predispose susceptible individuals through epigenetic programming, whereas postnatal factors—including excess weight gain, adiposity, insulin resistance, diet, physical inactivity, and other environmental exposures—may trigger disease expression or exacerbate its clinical phenotype [24, 45]. This evolving gene‐environment model provides a framework for understanding disease heterogeneity and identifies opportunities for earlier prevention and risk stratification in genetically susceptible individuals.

Together, these intersecting pathways—neuroendocrine, steroidogenic, metabolic, and epigenetic—constitute the complex and still incompletely understood pathophysiology of PMOS (Fig. 3).

### Metabolic dysfunction in PMOS: from insulin resistance to diabetes

In PMOS, the reduced insulin sensitivity arising from tissue‐specific defects in insulin receptor and post‐receptor signaling pathways is central to the metabolic dysfunction. Genome‐wide association studies have implicated insulin resistance as a risk factor in the development of PMOS [48, 49], and a polygenic risk score‐powered phenome‐wide association study has demonstrated substantial shared biological pathways between PMOS and a range of metabolic outcomes, particularly T2DM and obesity [50]. Insulin resistance and the resultant hyperinsulinemia contribute to both clinical disease expression and broader metabolic risk through multiple mechanisms.

Insulin resistance severity varies and is present independent of BMI, although overweight and obesity further exacerbate the risk [51, 52]. This translates into an increased likelihood of developing impaired glucose tolerance (IGT) and T2DM. A pooled analysis of 41 studies incorporated into the updated 2023 International Guideline for the assessment and management of PMOS reported odds ratios of 3.2, 3.9, and 2.9 for impaired fasting glucose, IGT, and T2DM, respectively [2]. However, these risks are not uniform across all women with PMOS. A 2018 systematic review demonstrated substantial ethnic variation in IGT risk, with 5.2‐, 4.4‐, and 2.6‐fold increased odds among Asian, North/South Americans, and European women, respectively. Similarly, body weight exerts a significant influence, with lean‐matched and overweight/obesity‐matched groups exhibiting 4.4‐ and 2.5‐fold higher odds of IGT, respectively [53]. A family history of T2DM also increases risk: In a retrospective study of 408 women with PMOS, a family history of T2DM significantly increased the likelihood of IGT or T2DM, as well as the probability of an adverse metabolic profile, even among those with normal glucose tolerance [54]. Importantly, the increased risk of T2DM is also present in lean women with PMOS, and women with PMOS are diagnosed at an earlier age [55]. Collectively, these findings establish T2DM as one of the most robust and clinically important long‐term complications of PMOS, supporting systematic metabolic surveillance across the life course. In light of these findings, the updated 2023 International Guideline recommends glycemic surveillance for women with PMOS at least every 3 years, with shorter screening intervals advised for individuals at higher metabolic risk (e.g., women with a history of gestational diabetes, a family history of T2DM, elevated BMI, or higher‐risk ethnicity) [2]. The guideline also identifies the 75‐g oral glucose tolerance test (OGTT) as the preferred method for assessing glycemic status, based on evidence from a systematic review and meta‐analysis demonstrating its superiority over fasting plasma glucose and HbA1c for diagnosing T2DM in this population [56]. Nevertheless, this recommendation is graded as weak, in part because of the practical challenges associated with the OGTT, including cost, time burden, inconvenience, and limited accessibility, underscoring the need for further research into alternative diagnostic strategies.

#### Metabolic syndrome and metabolic dysfunction‐associated steatotic liver disease (MASLD)

Women with PMOS have a higher risk of metabolic syndrome, driven by an increased prevalence of its key components, including central obesity, insulin resistance, dyslipidemia, and hypertension. Evidence from a systematic review and meta‐analysis of 59 studies shows that the elevated prevalence of metabolic syndrome in PMOS is primarily linked to obesity and other metabolic abnormalities, rather than hyperandrogenism [8]. Metabolic dysfunction‐associated steatotic liver disease (MASLD), which is closely tied to metabolic syndrome, is likewise more common among women with PMOS, with a suggested pooled prevalence of 43% in women with PMOS [57]. Meta‐regression analyses further identify metabolic syndrome, insulin resistance, and biochemical markers of hyperandrogenism as significant risk factors for MASLD in this population [57].

### Cardiovascular disease risk: understanding the connections

CVD accounts for approximately 35% of deaths among women globally, exceeding mortality from all cancers combined [58]. Despite this burden, CVD in women remains underdiagnosed, undertreated, and underrepresented in clinical research [59]. Traditional cardiovascular risk assessment tools—developed primarily in male populations—may fail to capture sex‐specific risk factors, such as PMOS, that emerge earlier in the female lifespan. The metabolic phenotype of PMOS promotes accelerated CVD development, and with the high prevalence of the condition, special emphasis should be placed on preventive studies and resources for managing risk factors leading to CVD [2, 60].

#### Cardiovascular risk factors in PMOS

Women with PMOS demonstrate a markedly elevated prevalence of traditional cardiovascular risk factors—including childhood weight gain, obesity, insulin resistance and compensatory hyperinsulinemia (discussed above), atherogenic dyslipidemia, and chronic low‐grade inflammation. All these risk factors emerge decades earlier than in the general female population. Understanding this clustering of risk factors is essential for early identification and intervention.

#### Dyslipidemia

Atherogenic dyslipidemia represents another cardinal feature of PMOS, affecting 60%–70% of women with the condition [61]. The lipid profile typically demonstrates elevated triglycerides, reduced high‐density lipoprotein (HDL) cholesterol, and increased small dense LDL particles with enhanced atherogenic potential [61, 62]. The dyslipidemia in PMOS results from multiple mechanisms, including hepatic overproduction of very‐low‐density lipoprotein (VLDL) particles, impaired lipoprotein lipase activity, enhanced cholesteryl ester transfer protein activity, and altered adipose tissue lipolysis driven by insulin resistance and hyperandrogenism [63]. A recent study found that women with PMOS had a classic atherogenic lipid pattern, characterized by increased VLDL cholesterol and triglycerides and reduced HDL cholesterol, but with less unfavorable lipoprotein subclass distribution. Notably, antiandrogenic regimens containing ethinyl‐estradiol (EE) increased triglyceride‐rich lipoproteins and medium LDL and small HDL particles compared with metformin, whereas small LDL particles increased only with EE/desogestrel plus spironolactone. Whether these changes translate into increased long‐term CVD risk remains unknown [64].

#### Hypertension

Hypertension occurs 2–3‐fold more frequently in women with PMOS compared to age‐matched controls. In a Finnish population‐based study, 30% of PMOS cases reported hypertension compared to 18% in non‐PMOS counterparts [65]. The rise in antihypertensive medication use increased dramatically between ages 31 and 46 years; from 3.9% to 21.5% in PMOS and from 1.8% to 15.5% in controls. The data also revealed that in many cases, hypertension was suboptimally treated among women with PMOS at age 46, as the gap between diagnosis and medication in non‐PMOS women was 2.7%, whereas among PMOS cases, it was 8.6% [65]. Indeed, blood pressure elevation in PMOS often begins in the reproductive years rather than developing exclusively in midlife or postmenopause. Mechanisms driving hypertension include increased sympathetic nervous system activation, enhanced renin–angiotensin–aldosterone system activity, endothelial dysfunction with reduced nitric oxide bioavailability, and sodium retention mediated by hyperinsulinemia [66, 67]. The blood pressure elevation seems to be mediated through adiposity rather than BMI [68].

#### Weight gain, obesity, and body composition

Women with PMOS exhibit a higher prevalence of overweight, obesity, and central obesity, with relative risks of 1.95, 2.77, and 1.73, respectively, compared to women without PMOS [69]. Bidirectional Mendelian randomization studies indicate a causal effect of increased body weight on the development of PMOS, whereas PMOS itself does not appear to causally influence body weight [70]. Body weight therefore represents an important and potentially modifiable risk factor in women with PMOS who are overweight or obese. Weight gain exacerbates both the clinical manifestations of PMOS and its cardiometabolic consequences. Evidence from large‐scale UK electronic health record studies shows that weight gain is an independent predictor of progression to T2DM and cardiovascular events; notably, a 1% increase in BMI is associated with a 2% increase in T2DM risk [7, 71]. Consequently, lifestyle intervention—comprising structured exercise, behavioral strategies, and a balanced diet—remains central to PMOS management, with demonstrated benefits for metabolic health, including improvements in central adiposity and lipid profile.

Central obesity affects 40%–80% of women with PMOS, depending on the population and diagnostic criteria, with preferential accumulation of visceral adipose tissue conferring particularly elevated metabolic and cardiovascular risk [24]. Population‐based studies reveal that there is a BMI rise in PMOS already in childhood, with early adiposity rebound, that represents the first adiposity‐related event during a lifespan [72]. Thereafter, the weight gain remains higher among PMOS cases, being most striking between ages 14 and 31, and seems to be more pronounced with the hyperandrogenic PMOS phenotype [62]. Truncal fat accumulation is a known trait of PMOS, and waist and neck circumference measurements should be considered part of routine clinical practice, given their accuracy in assessing metabolic burden [73, 74].

#### Inflammatory and thrombotic markers

Chronic low‐grade inflammation characterizes PMOS, with implications for atherogenesis and plaque instability [24]. C‐reactive protein levels are elevated 2–3‐fold compared to weight‐matched controls, with concentrations frequently exceeding 3 mg/L—a threshold associated with high cardiovascular risk [75]. Pro‐inflammatory cytokines, including interleukin‐6, interleukin‐18, and tumor necrosis factor‐alpha, are elevated in PMOS, promoting endothelial activation, monocyte recruitment, and foam cell formation [76]. Women with PMOS also exhibit prothrombotic tendencies, evidenced by elevated plasminogen activator inhibitor‐1, increased platelet activation markers, and enhanced thrombin generation, potentially contributing to acute cardiovascular event risk [77]. The inflammatory state appears partly independent of obesity, as lean women with PMOS maintain elevated inflammatory markers, suggesting that hormonal dysregulation and insulin resistance directly activate inflammatory pathways [78].

#### Endothelial dysfunction and subclinical atherosclerosis

Endothelial dysfunction—representing the earliest detectable stage of atherosclerotic CVD—manifests consistently in PMOS populations [79, 80]. Flow‐mediated dilation (FMD), a noninvasive marker of endothelial health, demonstrates a 20%–30% reduction in women with PMOS compared to controls, indicating impaired nitric oxide‐mediated vasodilation. Indeed, besides FMD, women with PMOS have subclinical CVD as measured by arterial stiffness, coronary artery calcium scores, carotid intima‐media thickness, and visceral and epicardial fat [79]. Coronary artery calcification, detected via computed tomography, appears more frequently and extensively in premenopausal women with PMOS than in controls, and the findings seem to be linked to hyperandrogenism [80].

#### Cardiovascular disease and major adverse cardiovascular events risk in PMOS

Multiple large‐scale population‐based cohort studies have conclusively demonstrated elevated CVD risk in women with PMOS across diverse healthcare systems and geographic regions [7, 81]. In a Finnish population‐based study, a significant increase in MACE was observed in both the Rotterdam‐PMOS and NIH‐PMOS groups, with rates of 2.33 (1.26–4.30) and 2.47 (1.18–5.17), respectively [82]. Alarmingly, the cumulative hazard curves for both diagnostic categories diverged at age 35. The early incline in risk was also observed in a large UK‐based study [7]. Similarly, a Danish study examined 18,112 Danish women with PMOS versus 54,336 matched controls, revealing incident CVD HR 1.71 (95% CI, 1.56–1.88), with particularly elevated risks for myocardial infarction (HR 1.78) and ischemic heart disease (HR 1.73) [83]. Obesity, diabetes, infertility, and previous use of oral contraceptives were associated with increased risk of development of CVD in PMOS [83]. In a large Nordic population‐based cohort of nearly 130,000 women with PMOS from Finland, Denmark, and Sweden, pooled estimates showed an increased risk of CVD (HR 1.27 [1.18; 1.36]) [84]. Moreover, MACE (1.21 [1.10; 1.34]) and deep venous thrombosis (1.30 [1.27; 1.33]) were elevated as well as CVD risk in PMOS women with BMI <25 kg/m^2^ (1.40 [1.26; 1.55]). These contemporary population‐based studies represent an important advance, moving the field beyond associations with cardiovascular risk factors to convincing evidence that PMOS is associated with increased cardiovascular events. All these findings call for early risk identification, diagnosis, and treatment.

PMOS represents an early‐life marker of CVD in women, with population‐based evidence demonstrating increased risk of MACE. The constellation of metabolic risk factors emerges decades before clinical events, highlighting an extended window for prevention and the need for early identification and targeted intervention.

### Reproductive impact of PMOS: fertility and pregnancy complications

In PMOS, high follicle and oocyte counts and elevated AMH levels lead to a disrupted endocrine milieu causing OD [85, 86]. Anovulation is the most common reason for female infertility, and among women with anovulation, the majority is due to PMOS [87]. Conversely, 90%–95% of women diagnosed with PMOS experience OD [88, 89]. Spontaneous ovulations occur, but it may take longer to achieve pregnancy. In the Nordic countries, women with PMOS have at least one child as often as other women [90, 91], whereas a recent Australian study shows greater nulliparity when assessing women close to the end of their reproductive lifespan [92]. Women with PMOS also generally give birth at a later age than women without PMOS, and more than half of women with PMOS have undergone fertility treatments [92]. This underscores the importance of early diagnosis and proper information to patients regarding family planning to achieve the desired family size [92, 93].

Because overweight and obesity adversely impact fertility and fertility treatment outcomes, a healthy lifestyle to optimize prevention of weight gain is vital. Overweight women have lower pregnancy rates and an increased risk of miscarriage compared to women with normal weight [94]. Furthermore, overweight women often respond more poorly to ovarian stimulation and produce poorer quality oocytes and embryos [95]. Preconception care with age‐appropriate education on how to optimize reproductive health, including education on healthy lifestyle choices and weight management, is recommended both for adolescents and young women with PMOS [2]. However, the role of weight loss, the strategy, extent, duration, and impacts in those with obesity are not yet clear. Once obesity is established, it is largely considered a non‐modifiable risk factor when applying lifestyle alone, and medical and surgical therapies may be needed to reduce weight, aligned with population guidelines. Yet, there are no trials of these strategies on live birth, and more research is definitely needed. This also emphasizes the role of early diagnosis and a healthy lifestyle for prevention.

If pregnancy is not desired, providing contraception should not be forgotten, as fertility in women with PMOS is difficult to predict.

Women with PMOS are more likely to experience pregnancy complications, including gestational diabetes mellitus (GDM) (OR 2.4), preeclampsia (OR 2.3), miscarriage, hypertension, induction of labor, and cesarean section [96]. The increased pregnancy risks are independent but exacerbated by higher maternal age and higher preconception BMI in women with PMOS compared to those without [96]. Other underlying factors include impaired glucose metabolism, excessive androgen production, and chronic mild inflammation [97]. These observations reinforce the concept that reproductive outcomes are closely linked to the broader metabolic and endocrine disturbances that characterize PMOS, highlighting pregnancy as an important window for identifying women at increased long‐term cardiometabolic risk [98, 99].

The combination of PMOS and GDM poses significant risks also to the fetus. A case‐controlled study found an increased risk for neonatal hypoglycemia in women with both PMOS and GDM compared to those with GDM alone, with an incidence of 17% and 5%, respectively [100]. Moreover, offspring born to mothers with PMOS have an increased risk of being born prematurely and developing neonatal jaundice and respiratory complications compared to those of mothers without PMOS [101, 102].

Because of the increased risks for adverse pregnancy outcomes, the International Evidence‐Based PMOS Guideline recommends considering pregnancies of women with PMOS as risk pregnancies with tailored, individual monitoring and early intervention when needed [2].

### Current and emerging treatment strategies: lifestyle, pharmacotherapy, and beyond

Pharmacological treatment in PMOS should be individualized and guided by the predominant clinical features, including metabolic dysfunction, hyperandrogenism, and reproductive goals.

Although current management remains largely symptom‐directed, increasing emphasis is being placed on early identification and treatment of metabolic dysfunction to reduce long‐term cardiometabolic risk. Although lifestyle management is strongly recommended in PMOS, especially for prevention of excess weight gain, it is often insufficient for sustainably reducing weight, metabolic, and endocrine features. There is a range of evidence‐based but off‐label pharmacological therapies used to treat symptoms of PMOS. Current pharmacological management of PMOS—including the principal indications, main effects, and key clinical considerations for commonly used therapies—is summarized in Table 3. Personalized treatment according to the individual's predominant clinical manifestations, metabolic risk profile, and reproductive goals is recommended.

**Table 3:** Current pharmacological management of polyendocrine metabolic ovarian syndrome (PMOS).

Intervention	Main indication	Main effects	Key considerations
Lifestyle intervention	All women	Improves metabolic health, weight management, and quality of life	Cornerstone of management throughout the life course
Metformin	Metabolic dysfunction, insulin resistance	Improves insulin sensitivity and glycemic outcomes	Recommended particularly in adults with BMI ≥25 kg/m^2^
GLP‐1 receptor agonists	Obesity	Substantial weight loss; improves cardiometabolic risk factors	Evidence in PMOS is emerging and remains more limited than in the general population; avoid during pregnancy
Bariatric surgery	Severe obesity	Improves weight, diabetes, ovulation, and fertility	Consider in severe obesity after multidisciplinary assessment
Combined oral contraceptive pill	Hyperandrogenism, menstrual dysfunction	Improves hirsutism, acne, and cycle regularity; provides endometrial protection	First‐line treatment for reproductive manifestations
Anti‐androgens	Persistent hirsutism (any clinical hyperandrogenic feature)	Reduces androgenic symptoms	Use with effective contraception
Inositol	Selected women with metabolic features	Modest biochemical improvements	Inferior to metformin; may be considered with informed consent

Abbreviation: BMI, body mass index.

#### Metabolic management

Metformin, an insulin sensitizer originally developed for T2DM, is extensively used in PMOS off‐label, in line with International Guideline recommendations. A recent meta‐analysis demonstrated a modest but significant reduction in BMI (mean difference −0.53 kg/m^2^, 95% CI −0.95 to −0.12) together with improvements in insulin resistance and fasting glucose compared with placebo [103]. Based on this, the International Evidence‐Based PMOS Guideline recommends metformin for adults with PMOS and a BMI ≥25 kg/m^2^ for anthropometric and metabolic outcomes, including insulin resistance, glucose, and lipid profiles [2]. However, evidence from a recent RCT suggests that metformin may not be superior to combined oral contraceptive pill (COCP) in preventing development of metabolic syndrome, as no significant difference was observed between treatment groups [104].

The optimal dose remains uncertain; current expert opinion suggests a maximum daily dose of 2.5 g/day for adults and 2.0 g/day for adolescents [2, 103]. An ongoing RCT is evaluating the optimal dosing of metformin in women with PMOS [105].

Although metformin contributes to modest weight loss, GLP‐1 receptor agonists (GLP‐1 RAs) appear to be more effective in this regard [106, 107]. Gastrointestinal side effects are common with metformin but are even more common when using GLP‐1 RAs [106].

Inositol modulates insulin signaling pathways and is therefore considered an insulin sensitizer. A recent comprehensive systematic review, which excluded trials with concerns regarding research integrity, demonstrated that inositol may produce modest improvements in biochemical parameters, including hyperandrogenism and insulin resistance, but did not significantly improve clinical outcomes and was inferior to metformin [108]. However, quality of evidence for many outcomes was low or very low and should be interpreted with caution. According to the International Evidence‐Based PMOS Guideline, inositol may be considered with informed consent, particularly in situations where access to metformin or more effective medications is limited, while acknowledging limited evidence [2].

#### Weight management

Anti‐obesity pharmacological therapies represent a potentially valuable option for improving cardiometabolic risk and clinical symptoms in PMOS.

Although GLP‐1 RAs are well established as effective treatments for improving body weight, lipid profile, and glycemic control in individuals with T2DM [109], the evidence in PMOS remains limited. A recent systematic review of 11 randomized controlled trials demonstrated a significant reduction in BMI of 1.38 kg/m^2^ (95% CI 0.38–2.39 kg/m^2^) in women with PMOS and overweight or obesity [107], but uncertainty persists regarding metabolic, reproductive, and psychological outcomes due to low‐quality data. However, most previous trials have been limited by their short duration, small sample sizes, and reliance on older GLP‐1 RA formulations that are considerably less potent than agents such as semaglutide or tirzepatide. There is therefore an urgent need for large, rigorously designed clinical trials, with accompanying health economic evaluation, to address this significant area of unmet clinical need. Notably, GLP‐1 RAs are contraindicated during pregnancy, and contraception use is recommended while using them [110].

In the cases of severe obesity, where pharmacological treatment is insufficient, the possibility of bariatric surgery should be considered. Bariatric surgery improves weight loss, hypertension, diabetes (prevention and treatment), hirsutism, irregular menstrual cycles, ovulation, and pregnancy rates in women with PMOS [111]. This is supported by findings from the BAMBINI clinical trial, in which substantial weight loss induced by bariatric surgery was more effective than standard medical care in restoring ovulation [112]. Women undergoing bariatric surgery should be informed that fertility might increase rapidly. Ideally, preconception planning with a delay in conception until at least 12 months post‐surgery and an assessment of micronutrient status before attempted pregnancy is recommended [113].

#### Management of hyperandrogenism and menstrual dysfunction

Treatment of hyperandrogenic symptoms and menstrual irregularity primarily targets suppression of ovarian androgen production and regulation of the menstrual cycle.

COCPs are recommended in PMOS for management of hormonal disturbances, irregular menstrual cycles, endometrial protection, contraception, and hyperandrogenic features (hirsutism and acne) [2, 114]. Although evidence on COCPs containing natural estrogens (estradiol (E2) or estetrol (E4)) in PMOS is limited, preparations containing low‐dose EE are generally recommended [115]. Regarding the progestin component, a recent meta‐analysis found that cyproterone acetate and fourth‐generation progestins are the most effective for reducing hyperandrogenism. However, due to an increased risk of venous thrombosis, cyproterone acetate is recommended only as second‐line treatment [115]. Evidence on progestin‐only pills (POPs) in PMOS is limited, but they may be considered in women with contraindications to COCPs. Both COCPs and POPs can be used for endometrial protection, thereby lowering the risk of hyperplasia and endometrial cancer [2, 24].

Current evidence does not support the use of anti‐androgens over COCPs for management of hyperandrogenic features in PMOS [116]. However, anti‐androgens may be considered for the treatment of hirsutism in combination with effective contraception if there is suboptimal response after at least 6 months of COCP therapy [2]. COCPs and cosmetic treatments are considered first‐line therapy for hirsutism in women with PMOS [2]. Traditional hair removal methods, such as shaving, waxing, and epilation, provide only temporary results. In contrast, selective thermolysis and laser therapy offer long‐lasting reduction and should be considered, particularly for facial hirsutism.

Hirsutism is frequently identified by patients as a key concern due to its significant impact on psychological well‐being. Women with PMOS may require a higher number of laser treatments compared with those with idiopathic hirsutism to achieve hair reduction, and treatment is relatively ineffective for those with blond or grey hair [2].

## Conclusion: redefining PMOS

PMOS has been renamed to reflect major advances in our understanding of the condition, recognizing that there is no increase in pathological ovarian cysts and that the disorder extends far beyond the ovary to encompass endocrine, metabolic, cardiovascular, reproductive, dermatological, and psychological features. Overall, it should be recognized as a common, multisystem disorder with significant metabolic, reproductive, dermatological, and psychological implications across the lifespan.

Recent advances have transformed the field, with large population‐based studies demonstrating increased risks of Type 2 diabetes and MACE, whereas mechanistic studies have strengthened the evidence that insulin resistance represents a central link between reproductive dysfunction and long‐term cardiometabolic disease. New frontiers recognize the diversity of PMOS and seek to progress clinically meaningful ways to define this heterogeneity and to understand natural history. Key priorities include advancing precision medicine based on genetic, endocrine, and metabolic profiles and developing validated risk prediction tools to guide personalized management and earlier preventive strategies. Clinical research priorities have been defined in co‐produced global and UK multistage processes [22, 117]. These include refining diagnostic thresholds, cardiometabolic risk stratification, and long‐term prevention and management pathways, including optimal obesity management.

Although management remains largely guided by the predominant clinical manifestations, increasing emphasis should be placed on early recognition of metabolic dysfunction and cardiometabolic risk across the life course. As we adopt the new name, future directions will depend on continued global collaboration, robust evidence synthesis, and patient‐centered approaches that reflect the complexity of this lifelong condition.

## Author contributions

**Maria Forslund**: Conceptualization; project administration; writing—original draft; writing—review and editing. **Johanna Melin**: Writing—original draft; writing—review and editing. **Anju E. Joham**: Writing—original draft; writing—review and editing. **Terhi T. Piltonen**: Writing—original draft; writing—review and editing. **D. Aled Rees**: Writing—original draft; writing—review and editing. **Helena J. Teede**: Conceptualization; writing—original draft; writing—review and editing. All authors contributed to manuscript development, critically revised the manuscript for important intellectual content, and approved the final version.

## Conflict of interest statement

The authors declare no conflicts of interest.
